# No‐shows to hidradenitis suppurativa clinic visits: Patient perspectives

**DOI:** 10.1002/ski2.322

**Published:** 2023-12-10

**Authors:** Natalie M. Fragoso, Rahul Masson, Terri Shih, Tiffany Mayo, Ginette Okoye, Vivian Y. Shi, Jennifer L. Hsiao

**Affiliations:** ^1^ Department of Dermatology Dartmouth Hitchcock Medical Center Lebanon New Hampshire USA; ^2^ Keck School of Medicine University of Southern California Los Angeles California USA; ^3^ David Geffen School of Medicine University of California Los Angeles Los Angeles California USA; ^4^ Department of Dermatology University of Alabama at Birmingham Birmingham Alabama USA; ^5^ Department of Dermatology Howard University Washington District of Columbia USA; ^6^ Department of Dermatology University of Arkansas for Medical Sciences Little Rock Arkansas USA; ^7^ Department of Dermatology University of Southern California Los Angeles California USA

## Abstract

**Background:**

Hidradenitis suppurativa (HS) is a debilitating chronic inflammatory skin condition that typically requires consistent care. Contributory factors to why patients with HS miss their clinic appointments have not been investigated.

**Objectives:**

This article seeks to characterise reasons that HS patients do not keep their appointments and identify strategies to minimise no‐show rates and improve delivery of care to HS patients.

**Methods:**

An anonymous survey was distributed to online HS support groups.

**Results:**

Of the 254 respondents, 18.9% reported ever missing an appointment for HS. Common reasons for missing an appointment include: patient was experiencing an HS flare (72.9%), prior poor experience with a healthcare provider (54.2%) or healthcare staff member (37.5%), embarrassment of condition (41.7%), and distrust that the appointment would help with management of HS (39.6%). Respondents who were non‐White, who were disabled, or who had lower socioeconomic status were more likely to have missed an appointment (*p* < 0.05).

**Conclusions:**

This study highlights areas where dermatologists may help improve appointment attendance, including encouraging patients to seek care during flares and striving to optimise the patient experience.

1

**What is already known about this topic?**

- Missing clinic appointments can negatively impact care delivery, however, the reasons that hidradenitis suppurativa (HS) patients miss appointments have not been well‐studied.

**What does this study add?**

- This study found that 18.9% of respondents reported having ever missed an HS clinic appointment.
- Top reasons for missing an HS clinic visit included: HS flare, prior poor experience with a healthcare provider or staff member, embarrassment, and belief that the appointment would not be helpful.
- Patients who were non‐White, disabled, or with a lower household income were more likely to miss appointments.

## 2 INTRODUCTION

Hidradenitis suppurativa is a chronic inflammatory skin condition that often severely impacts patient quality of life.^1^ Missed clinic visits or “no‐shows” can hinder care delivery. Hidradenitis suppurativa patient perspectives regarding missed clinic visits are not well‐characterised. Understanding the reasons why HS patients miss visits can help dermatologists try to minimise no‐shows and deliver much‐needed care.

## 3 PATIENTS AND METHODS

An anonymous survey (Appendix S1) was distributed with group administrator permission through online HS support groups (Hope for HS, HS Connect, and HS Warriors) between August and October 2022 and respondents 18 years or older were eligible to participate. Data were analysed in R version 4.2.1 and a *p*‐value of <0.05 was considered significant. Multivariable logistic regression was used to examine the likelihood of missing an appointment based on gender (female VS other), disability (disabled VS not disabled), race (non‐White VS White), educational level (completed high school/higher education VS less than high school), insurance status (public VS non‐public), primary HS provider (dermatologist VS non‐dermatologist), Hurley stage (stage II/III VS stage I), duration of HS, and household income (≤$50,000 VS >$50,000 annually) while controlling for all other variables above. Of note, the data analysis was limited by a small sample size and a non‐normal distribution. Patients self‐reported their Hurley stage based on descriptions provided of each stage in the survey. Any missed appointment for HS was considered a no‐show.

## 4 RESULTS

**FIGURE 1:**
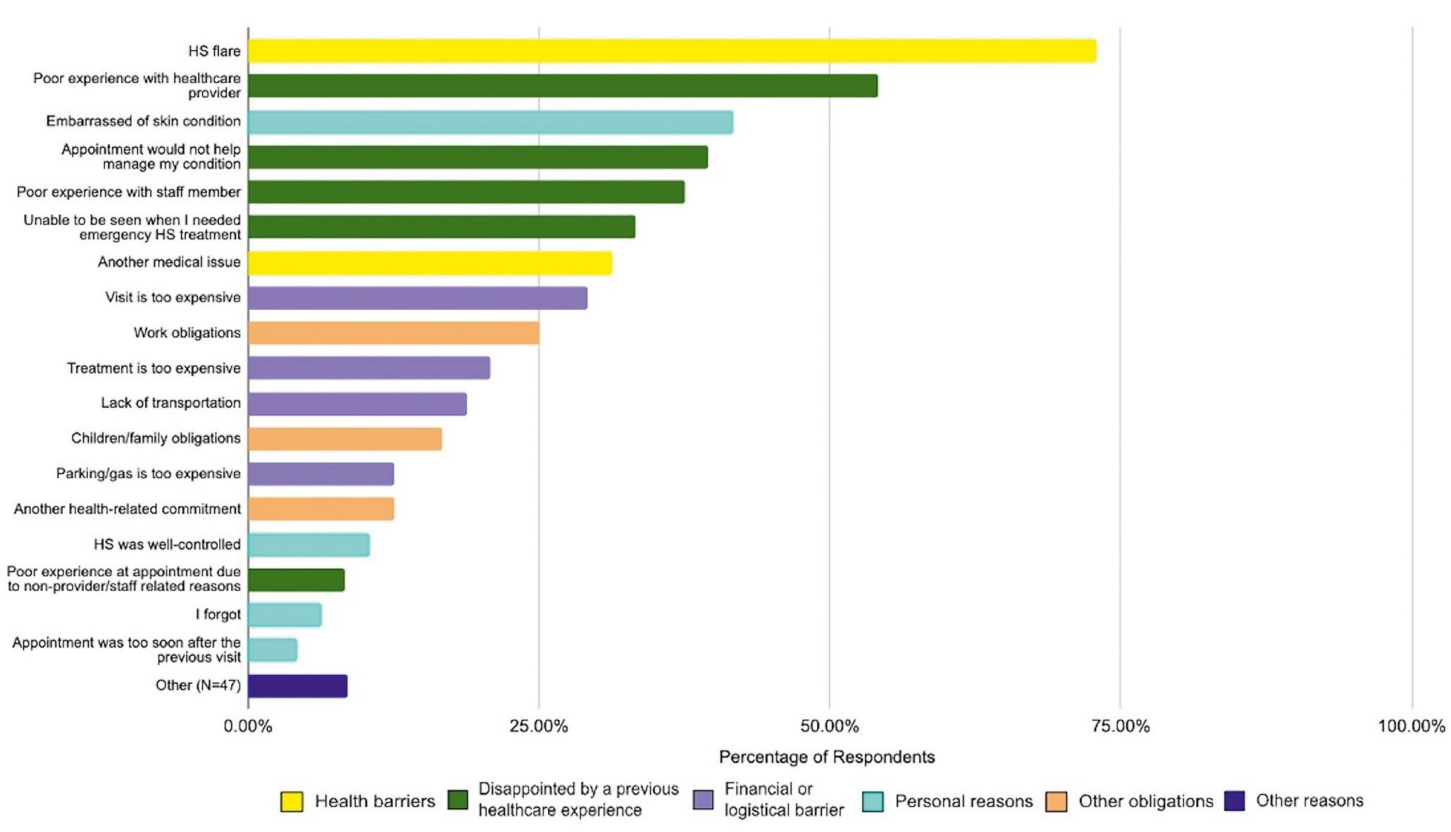
Participant reasons for missed clinic appointments.

254 participants responded to the survey; their demographics are listed in Table 1. Forty‐eight (18.9%) participants had previously missed an appointment with their primary HS provider (provider who has seen them for >50% of their visits). Overall, top categories of reasons for missing appointments include health barriers (85.4%) and disappointment with a previous healthcare experience (66.7%), followed by financial or logistical barriers (56.3%), personal reasons (52.1%), and other obligations (37.5%). The top five reasons for missing an appointment were: HS flare (72.9%), poor experience with a healthcare provider (54.2%), embarrassment of skin condition (41.7%), distrust that the appointment would help with management of HS (39.6%), and poor experience with a healthcare staff member (37.5%) (Figure 1). A minority of patients reported forgetting the appointment (6.3%) or that the visit was too soon after the previous one (4.2%). Participant‐reported strategies that would help them keep appointments included more flexible scheduling (37.5%), allowing online cancellations (35.4%), offering telehealth appointments (27.1%), sending reminder texts or emails (27.1%), and helping with transportation coordination (12.5%). Author recommendations to minimise no‐shows are summarised in Table 2.

**TABLE 1:** Respondents' demographics.

Demographic characteristics	N (%)
Age, mean ± SD (range) (*n* = 251)	41.5 ± 11.8 (18–80)
Age at HS symptom onset, mean ± SD (range) (*n* = 252)	20.8 ± 11.2 (3–68)
Age at HS diagnosis, mean ± SD (range) (*n* = 234)	32.1 ± 12.1 (5–74)
Duration of HS, mean ± SD (range) (*n* = 251)	20.9 ± 12.9 (0–62)
Gender (*n* = 253)	
Female	218 (86.2%)
Male	32 (12.6%)
Other^a^	3 (1.2%)
Race/Ethnicity (*n* = 254)	
White	196 (77.2%)
Black	19 (7.5%)
Hispanic/Latino	17 (6.7%)
Bi‐ or multi‐racial	13 (5.1%)
Asian/Pacific Islander	5 (2.0%)
Other^b^	4 (1.6%)
Country of Residence (*n* = 248)	
United States	144 (58.1%)
Ireland	53 (21.4%)
Canada	19 (7.7%)
UK	17 (6.9%)
Other^c^	15 (6.0%)
Hurley stage (*n* = 254)	
Stage I	32 (12.6%)
Stage II	133 (52.4%)
Stage III	89 (35.0%)
Highest education level (*n* = 254)	
Less than high school	3 (1.2%)
High school graduate	39 (15.4%)
Some college	79 (31.1%)
Associates degree	28 (11.0%)
Vocational training	16 (6.3%)
Bachelor's degree	50 (19.7%)
Master's degree	27 (10.6%)
Doctorate or other professional degree	8 (3.1%)
Prefer not to say	4 (1.6%)
Annual household income (*n* = 253)	
<$30,000	50 (19.8%)
$30,001‐$50,000	50 (19.8%)
$50,001‐$75,000	47 (18.6%)
$75,001‐$100,000	37 (14.6%)
>$100,000	35 (13.8%)
Prefer not to say	34 (13.4%)
Employment status (*n* = 254)	
Currently employed (full‐time)	111 (43.7%)
Currently employed (part‐time)	20 (7.9%)
Disabled	43 (16.9%)
Unemployed	32 (12.6%)
Self‐employed	16 (6.3%)
Retired	13 (5.1%)
Student	12 (4.7%)
Prefer not to say	7 (2.8%)
Primary medical insurance (*n* = 251)	
Private	123 (49.0%)
Public	78 (31.1%)
No insurance	44 (17.5%)
VA	6 (2.4%)
Diagnosed with HS by a healthcare provider (*n* = 253)	236 (93.3%)
Managed at an HS specialty clinic (*n* = 254)	48 (18.9%)
Main HS provider (*n* = 254)	
Dermatologist	150 (59.1%)
Primary care doctor	69 (27.2%)
Surgeon	7 (2.8%)
Obstetrician/gynaecologist	7 (2.8%)
Emergency medicine doctor	6 (2.4%)
Other^d^	6 (2.4%)
I have never seen a healthcare provider for my HS	9 (3.5%)
Mode of travel to HS Provider's office (*n* = 254)	
I drive myself	166 (65.4%)
A friend or family member drives me	61 (24.0%)
I take the bus	9 (3.5%)
I take the train or subway	5 (2.0%)
I use a medical transportation service	1 (0.4%)
Other^e^	12 (4.7%)
Travel time to appointment with HS provider (minutes) (*n* = 253)	
0–10	43 (17.0%)
11–30	107 (42.3%)
31–60	62 (24.5%)
61–120	28 (11.1%)
>120	13 (5.1%)

Abbreviations: HS, hidradenitis suppurativa; N, number; SD, standard deviation; UK, United Kingdom; VA, Veteran's Affairs.

^a^ “Trans non‐binary”, “non‐binary”, unspecified (*n* = 1 each).

^b^ “Mix”, Native American, North African, Indian (*n* = 1 each).

^c^ Netherlands, Australia, United Arab Emirates (*n* = 2 from each); Belgium, India, Morocco, Puerto Rico, Iran, South Africa, Ghana, Puerto Rico, New Zealand (*n* = 1 from each).

^d^ Alternative medicine doctor (*n* = 2), urgent care doctor (*n* = 1), none/unspecified (*n* = 3).

^e^ Taxi (*n* = 2), telehealth (*n* = 2), “I'm not being treated” (*n* = 1), myself or family (*n* = 1), unspecified (*n* = 6).

**TABLE 2:** Recommendations to reduce Hidradenitis suppurativa (HS) clinic visit no‐shows.

Reason for missed appointment	Recommendation for minimising future no‐shows
Health‐related barriers	Educate patients about treatment options for flares, including in‐office procedures
	Encourage patient to seek care during flares when needed
	Create a written action plan for patients with stepwise treatment approach based on flare severity
	Implement a clinic protocol for managing patient messages regarding flares
	For high‐risk medically complex patients who may have missed visit due to another medical issue, consider having staff call to help reschedule the visit
Disappointment with previous healthcare experience (physician and/or staff)	Prioritise developing rapport and trust throughout visit
	Utilise positive communication strategies that acknowledge and validate patient concerns and align with patient's values
	Avoid discussion of sensitive topics such as weight loss until rapport has been established
	Train office and clinic staff to engage in non‐judgemental and thoughtful interactions (e.g. placing chux pad on exam table to minimise patient embarrassment with drainage)
Financial or logistical barriers	Offer telehealth appointments for follow‐up visits where appropriate and if feasible
	Partner with colleagues to offer additional flexible scheduling when available
	Discuss patient assistance programs, savings programs, and find affordable compounding pharmacies to help minimise medication costs
Personal reasons (e.g. embarrassed of skin condition, HS was well‐controlled, patient forgot appointment, or appointment was too soon)	Solicit patient input regarding timing of next appointment
	Encourage patient to explore resources such as HS support groups to help mitigate embarrassment and build resilience
	Educate patient regarding waxing and waning nature of HS and importance of continuity of care to allow for timely medication adjustments
	Utilise appointment reminder services

Compared to respondents who were not disabled, respondents who reported that they were disabled were more likely (odds ratio (OR) 3.51, *p* = 0.007) to miss an appointment. Respondents with an annual household income of ≤50,000 per year were more likely to have missed an appointment compared to those who earned >$50,000 per year (OR 2.38, *p* = 0.04). Non‐White respondents were more likely to have missed a previous HS appointment compared to White respondents (OR 2.6, *p* = 0.03). There was no statistical difference in reporting missed appointments based on gender, educational level, insurance status, primary HS provider, duration of HS, or Hurley stage.

Of the 187 participants who had never missed an appointment, reasons for appointment adherence included motivation to manage their HS (75%), concern about potential HS flare (37.8%), and desire to avoid being charged a fee for missing the appointment (18.8%).

## 5 DISCUSSION

Nearly a fifth (18.9%) of respondents reported missing an HS appointment before. The most highly‐reported reasons for missing appointments in this study were due to HS flare, poor experience with a healthcare provider, and embarrassment of skin condition. This contrasts with a previous survey study in the literature of general dermatology patients at an academic dermatology clinic in the United States that found that top patient reasons for missed appointments included forgetting about the appointment, lack of appointment reminders, work, and transportation problems.^2^

Participants reported HS flares as the most common reason for missing an appointment. Potential contributing factors to this finding include the increased pain, physical debilitation, and/or fatigue that patients experience during flares that make travel difficult. Unfortunately, a missed appointment may mean a missed opportunity for needed treatment modifications, contributing to more flares in the future and giving rise to a vicious cycle. This highlights the importance of patient education regarding treatment options for HS flares, including oral antibiotics and in‐office procedures such as intralesional steroid injections, incision and drainage, and focal de‐roofing. Patients should also be educated that scheduling or keeping an appointment with their primary HS provider during a flare is vastly preferred over seeking acute care at an urgent care clinic or emergency room. Of note, over a third of patients reported keeping their appointments due to concern for flares, and in the authors' experience, a contingent of patients will call the clinic to be urgently added on to the clinic schedule because of a flare. In both cases, providing patients with a written action plan for flares can help patients self‐manage flares at home and also provide guidance on when to call the clinic.^3^

With two‐thirds of participants reporting a previous negative experience with a healthcare provider or staff member as a reason for missing an appointment, the impact of establishing a positive, empathetic provider‐patient relationship cannot be overstated. Strengthening this relationship can help counter the pervasive distrust of medical care that exists among HS patients,^1^ which was also captured in this survey. Physician communication strategies should be examined for opportunities to help lower this barrier.^4^ For example, patients may prefer that sensitive topics such as weight loss and sexual health be addressed at a later visit, after rapport has been developed.^5^ Further, all staff, starting from the front‐desk and extending to the back office, should be trained to facilitate patient‐centred and non‐judgemental interactions, especially given the embarrassment patients with HS have reported feeling regarding their skin condition.

Over half of respondents reported a financial or logistical reason for missing an appointment, including issues with transportation and concerns regarding the cost of the visit, transportation, and/or treatment. Offering telehealth visits to avoid transportation issues entirely may also be beneficial, especially as HS is associated with low socioeconomic status.^6^ Of note, patient and provider opinions on the effectiveness of telehealth appointments are mixed.^7^, ^8^ Importantly, requests for photographs to aid medical assessments during telehealth appointments should be handled delicately, given that HS tends to affect sensitive areas.^8^ Providers may consider offering telehealth visits after an in‐person consultation has been performed and a treatment plan initiated. Implementing appointment reminder services may also help prevent no‐shows. Particular care should be paid to addressing barriers to access to care for vulnerable populations such as patients with a racial minority background, patients who are disabled, and patients with lower income, who were also more likely to report missing an appointment in this study.

Limitations of this study include that participants were predominantly White and female, response bias, recall bias, and unknown response rate, limiting generalisability of the results. The percentage of patients who have missed an HS appointment in our study was 18.9%, which is nearly the same as a previous study that found a rate of 18.6% for missed medical dermatology appointments at a large academic dermatology department.^9^ One Denmark study found no difference in no‐show rates between patients seen in HS‐specific clinics when compared with patients seen in general dermatology outpatient clinics, though a limitation may be that HS patients are less likely to miss an appointment in an HS specialty clinic.^10^ In addition, patients who are engaged in online HS forums and were sent our survey may be more likely to seek care for their HS and attend appointments, which could have lowered the reported no‐show rate in our study.

In conclusion, dermatologists may help minimise HS appointment no‐shows by educating patients about options for treatment during flares, encouraging patients to attend appointments during flares, striving to develop rapport and build trust with patients throughout all aspects of their encounter, and offering telehealth appointments in appropriate circumstances. It is important for dermatologists to help overwrite the previous negative healthcare experiences that many patients with this highly‐stigmatised and under‐recognized condition have faced before they come through our clinic doors.

## CONFLICT OF INTEREST STATEMENT

VYS is on the board of directors for the Hidradenitis Suppurativa Foundation (HSF), is a stock shareholder of Learn Health and has served as an advisory board member, investigator, speaker, and/or received research funding from Sanofi Genzyme, Regeneron, AbbVie, Eli Lilly, Alumis, Novartis, SUN Pharma, LEO Pharma, Pfizer, Incyte, Boehringer‐Ingelheim, Aristea Therapeutics, Menlo Therapeutics, Dermira, Burt's Bees, Galderma, Kiniksa, UCB, WebMD, TARGET‐Pharmasolutions, Altus Lab, MYOR, Polyfin, GpSkin and Skin Actives Scientific. JLH is on the Board of Directors for the HS Foundation, has served as a consultant for Boehringer Ingelheim, Novartis, and UCB, and has served as a consultant and speaker for AbbVie. GO is on the board of the HSF and the Vaseline Healing Programme brand, is on advisory boards for Janssen, Novartis, AbbVie, Eli Lilly, Pfizer, and Sanofi Genzyme, and has served as a consultant for Unilever. NMF is an investigator for Acelyrin. All other authors have no conflicts of interest to declare.

## AUTHOR CONTRIBUTIONS

**Natalie M. Fragoso**: Conceptualisation (supporting); Methodology (equal); Writing – original draft (lead); Writing – review & editing (equal). **Rahul Masson**: Data curation (equal); Formal analysis (equal); Methodology (supporting); Writing – review & editing (equal). **Terri Shih**: Data curation (equal); Formal analysis (equal). **Tiffany Mayo**: Methodology (equal); Writing – review & editing (equal). **Ginette Okoye**: Methodology (equal); Writing – review & editing (equal). **Vivian Y. Shi**: Conceptualisation (supporting); Methodology (equal); Writing – review & editing (equal). **Jennifer L. Hsiao**: Conceptualisation (lead); Methodology (lead); Supervision (lead); Visualisation (lead); Writing – review & editing (lead).

## ETHICS STATEMENT

This study was approved under the exempt category by the institutional review board at the University of Southern California. The need for informed consent was waived by the institutional review board at the University of Southern California.

## Supporting information

Supplementary Material

## Data Availability

All data generated or analyzed during this study are included in this article. Further enquiries can be directed to the corresponding author.
