# Supplementary material for: No‐shows to hidradenitis suppurativa clinic visits: Patient perspectives
Source: Skin Health Dis. 2023 Dec 10;4(2):e322. doi: 10.1002/ski2.322 (PMC10988716; doi:10.1002/ski2.322)
Supplement: Supplementary file 1 — Supplementary Material [file SKI2-4-e322-s001.docx]

No-Shows to Hidradenitis Suppurativa Clinic Visits: Patient Perspectives Survey

1. What is your age?
   - Drop down menu
2. What is your gender?
   - Male
   - Female
   - Other (free text)
3. Which of the following best describes your race/ethnicity?
   - White
   - Black
   - Asian/Pacific Islander
   - Hispanic/Latino
   - Bi- or multi-racial
   - Other (free text)
4. Which country do you live in?

- Dropdown menu with country options

1. How severe (what Hurley stage) is your HS now? Please select one answer based on the worst site on your body.
   - Hurley stage 1: one or more abscesses or painful bumps WITHOUT tunnels under the skin or scars
   - Hurley stage 2: one or more widely separated recurring abscesses or painful bumps WITH tunnels under the skin and scarring
   - Hurley stage 3: multiple interconnected abscesses and tunnels under the skin across an entire body site
2. What is the highest level of education you have completed?
   - Less than high school
   - High school graduate (or equivalent)
   - Some college (1-4 years, no degree)
   - Vocational training
   - Associates degree
   - Bachelor degree
   - Master degree
   - Doctorate or other professional degree
   - Prefer not to say
3. What is your annual household income?
   - <$30,000
   - $30,001-$50,000
   - $50,001-$75,000
   - $75,001-$100,000
   - >$100,000
   - Prefer not to say
4. What is your employment status?
   - Currently employed (full-time)
   - Currently employed (part-time)
   - Self-employed
   - Student
   - Retired
   - Disabled
   - Unemployed
   - Prefer not to say
5. What is your primary type of medical insurance?
   - Private insurance
   - Public insurance (Medicare/Medicaid)
   - Veterans Affairs (VA) insurance
   - No insurance
6. What is your zip code?
   - Free text
7. How old were you when your HS symptoms began? (in years)
   - Dropdown menu (0-100 years old)
8. Have you been diagnosed with HS by a healthcare provider?

- Yes
- No

1. How old were you when were you diagnosed with HS? (in years)
   - Dropdown menu (0-100 years old)
2. Who is your main HS healthcare provider (**who you see for >50% of your HS related visits)**? Please select one best answer.
   - ​​Dermatologist
   - Primary care doctor (including internal medicine or family medicine doctor)
   - Pediatrician
   - Surgeon
   - Obstetrician/Gynecologist (OB/GYN)
   - Urologist (doctor specializing in conditions of kidneys, bladder and genital system)
   - Emergency medicine doctor
   - Rheumatologist (doctor specializing in joint and muscle conditions)
   - Infectious disease doctor
   - Psychiatrist, Counselor, Mental Health Provider
   - Naturopathic/ayurvedic/herbalist/Chinese medicine doctor
   - Other (free text)
   - I have never seen a healthcare provider for my HS
3. Are you currently managed at a HS specialty clinic? Examples of HS specialty clinics are listed in the link ([Hidradenitis Suppurativa Specialty Clinic Locations.docx](https://docs.google.com/document/d/1CkqZelZCh72iHwmAZOxNeiH7z2iKdq0q/edit?usp=sharing&ouid=103329263056656341805&rtpof=true&sd=true)) if you are unsure.
   - Yes
   - No
4. How do you typically travel to your main HS provider’s office (choose the main mode of transportation)?
   - I drive myself
   - A friend or family member drives me
   - I use a medical transportation service
   - I take the bus
   - I take the train or subway
   - I fly on an airplane
   - Other (free text)
5. How far do you have to travel from your home to get to your appointment with your main HS provider?
   - 0-10 minutes
   - 11-30 minutes
   - 31-60 minutes
   - 61-120 minutes
   - >120 minutes
6. Have you ever missed (not showed up to) an appointment with your main HS healthcare provider?
   - Yes
   - No
   - Not applicable

If No:

- 19) I have never missed an appointment for HS because (select all that apply):
  - - I am motivated to manage my condition
    - I am concerned about a potential flare
    - I do not want to be charged a fee for missing the appointment
    - Other (free text)

If yes:

- 20) What type of appointment was it? (select all that apply)
  - In-person appointment
  - Telehealth video appointment
  - Phone appointment
- 21) I have missed an **in-person appointment** for HS in the past because (select all that apply):
- Financial or logistical reasons:
  - The visit is too expensive
  - The medication/treatment is too expensive
  - Parking/gas is too expensive
  - I could not get transportation to the visit
- Health reasons:
  - I had a flare of my HS
  - Another medical issue prevented me from going to the appointment
- Other obligations:
  - I had to work
  - I had another health-related commitment
  - I had to take care of children/family
- Personal reasons:
  - I forgot
  - My HS was well-controlled so I did not need the appointment
  - The visit was too soon after the previous visit
  - I am embarrassed of my skin condition
- Disappointed by a previous visit:
  - I did not believe the appointment would help me manage my skin
  - I was not able to be seen when I needed emergency treatment for my HS
  - I had a bad experience at a medical appointment before:
    - Due to a healthcare provider (doctor, physician’s assistant (PA), nurse practitioner (NP))
    - Due to a staff member of the provider’s team
    - Due to another reason: (free text)
- Is there any other reason you have missed an **in-person** appointment in the past?
  - Yes (free text)
  - No
- Would any of the following actions from your healthcare team help prevent you from missing your appointment? (select all that apply)
  - Send a reminder text or email
  - Provide more flexible scheduling (for example, evenings, weekends)
  - Help coordinate transportation
  - Offer more telehealth appointments
  - Allow online cancellations
  - Other: (free text)
  - Unsure

22) Would you prefer to attend a telehealth visit compared to an in-person visit for your HS?

- Yes
- No
- Unsure
